# SARS-CoV-2 Genomes From Oklahoma, United States

**DOI:** 10.3389/fgene.2020.612571

**Published:** 2021-02-03

**Authors:** Sai Narayanan, John C. Ritchey, Girish Patil, Teluguakula Narasaraju, Sunil More, Jerry Malayer, Jeremiah Saliki, Anil Kaul, Pratul K. Agarwal, Akhilesh Ramachandran

**Affiliations:** ^1^Oklahoma Animal Disease Diagnostic Laboratory, College of Veterinary Medicine, Oklahoma State University, Stillwater, OK, United States; ^2^Department of Veterinary Pathobiology, College of Veterinary Medicine, Oklahoma State University, Stillwater, OK, United States; ^3^Department of Physiological Sciences, College of Veterinary Medicine, Oklahoma State University, Stillwater, OK, United States; ^4^Center for Health Sciences, Oklahoma State University, Tulsa, OK, United States; ^5^High-Performance Computing Center, Oklahoma State University, Stillwater, OK, United States

**Keywords:** SARS-CoV-2, nCoV-19, sequencing, mutations, Oklahoma

## Abstract

Genomic sequencing has played a major role in understanding the pathogenicity of severe acute respiratory syndrome coronavirus 2 (SARS-CoV-2). With the current pandemic, it is essential that SARS-CoV-2 viruses are sequenced regularly to determine mutations and genomic modifications in different geographical locations. In this study, we sequenced SARS-CoV-2 from five clinical samples obtained in Oklahoma, United States during different time points of pandemic presence in the state. One sample from the initial days of the pandemic in the state and four during the peak in Oklahoma were sequenced. Previously reported mutations including D614G in S gene, P4715L in ORF1ab, S194L, R203K, and G204R in N gene were identified in the genomes sequenced in this study. Possible novel mutations were also detected in the S gene (G1167V), ORF1ab (A6269S and P3371S), ORF7b (T28I), and ORF8 (G96R). Phylogenetic analysis of the genomes showed similarity to other SARS-CoV-2 viruses reported from across the globe. Structural characterization indicates that the mutations in S gene possibly influences conformational flexibility and motion of the spike protein, and the mutations in N gene are associated with disordered linker region within the nucleocapsid protein.

## Introduction

Toward the end of 2019, several individuals with signs of pneumonia reported to hospitals in Wuhan, the capital of Hubei Province in Central China. The etiological agent was identified to be a novel coronavirus (SARS-CoV-2/nCoV-19) on 7th January 2020 (Zheng, 2020). Human to human transmission was recorded around the same time (Nishiura et al., 2020). By the end of January 2020, WHO declared a “public health emergency of international concern” (Statement on the second meeting of the International Health Regulations 2005, 2020). As of December 22nd, 2020, a total of 78,263,502 confirmed patients and 1,722,307 deaths have been reported worldwide (Dong et al., 2020) and 2,65,620 registered cases and over 2,240 deaths in the state of Oklahoma, United States (OSDH-Covid-19 Tracker, 2020).

Numerous coronaviruses infecting different animal species including humans have been identified. SARS-CoV-2 is an enveloped positive-sense single-stranded RNA virus belonging to the genus *Betacoronaviru*s (subgenus *Sarbecovirus*) in the Coronaviridae family (order: Nidovirales). Based on genomic sequence analysis, it is reported to have originated from bats (Hu et al., 2015) and pangolins (Zhang et al., 2020a,b). Other previously identified coronaviruses known to infect humans include SARS-CoV-1, MERS-CoV, HCoV-NL63, HCoV-229E, HCoV-OC43, and HCoV-HKU1 (Graat et al., 2003; Van Elden et al., 2004; Mackay et al., 2012; Annan et al., 2013; Owusu et al., 2014; Corman et al., 2018).

Genomic sequence data is important in identifying, characterizing and understanding pathogens (Rambaut et al., 2008; Salipante et al., 2015). It can shed light on pathogenicity, virulence, drug/vaccine targets, mutation sites etc. and can also be critical in source attribution and determining microbial provenance (van Dorp et al., 2020). The first genome sequence of SARS-CoV-2 was made available on January 10th (GenBank ID: MN908947.3) (Wu et al., 2020). Multiple genomic sequences of SARS-CoV-2 from all over the world have since been deposited in public databases such as GenBank and GISAID (Global Initiative on Sharing All Influenza Data;^1^) (Elbe and Buckland-Merrett, 2017). This has facilitated extensive genomic studies leading to the identification of several mutations in the genome that can influence infectivity and virulence of the virus (Banerjee et al., 2020a; Daniloski et al., 2020; van Dorp et al., 2020).

The genome of SARS-CoV-2 is similar to many other pathogenic coronaviruses and has multiple genes that code for different proteins such as S gene (Surface glycoprotein), N gene (nucleocapsid phosphoproteins), M gene (membrane glycoprotein), E gene (envelope protein), and open reading frames, such as ORF1a, ORF1b ORF3a, ORF6, ORF7a, ORF7b, ORF8, and ORF10 (Khailany et al., 2020; van Dorp et al., 2020). The leading sequence of the viral genome is the sequence for ORF1ab, which encodes for multiple proteins including replicase polyproteins, non-structural proteins, papain-like proteinase, RNA-dependent RNA polymerase etc., which are essential for replication and survival in the host (Khailany et al., 2020). Though the exact function of ORF3a is yet to be clearly understood, it is believed to play a major role in viral release after replication in SARS-CoV-1 (Lu et al., 2009). ORF7 and ORF8 code for accessory proteins, the functions of which are yet to be clearly understood (Alam et al., 2020; Zhang et al., 2020c). Minimal roles of ORF7 and 8 in viral replication have been reported in SARS-CoV-1 alongside apoptosis stimulation of host cells (Liu et al., 2014).

The huge repository of sequence data in open-source databases such as the GenBank-NCBI and GISAID has facilitated the identification of numerous mutations and single nucleotide polymorphisms (SNPs) in the SARS-CoV-2 genome. SNPs in the genome that result in commonly reported non-synonymous mutations such as P4715L in ORF1ab, D614G in S gene, R203K, and G204R in N gene are some of the commonly reported. P4715L in ORF1ab is believed to play a major role in interaction with other proteins that regulate RNA dependent RNA polymerase (Pachetti et al., 2020). D614G (Aspartate to Glycine) mutation in the S gene has been reported to result in increased transduction into human epithelial cells (Daniloski et al., 2020). N gene mutations R203K and G204R are believed to increase viral fitness, survival and adaptation to humans (Leary et al., 2020).

In this study, we sequenced the genome of SARS-CoV-2 from 5 human clinical samples received at the Oklahoma Animal Disease Diagnostic Laboratory (OADDL) at various times during the SARS-CoV-2 pandemic in Oklahoma. Multiple mutations were detected in the genomic sequences including those already reported as well as previously unreported mutations.

## Materials and Methods

This study was approved by the Institutional Review Board (Application number: IRB-20-357) at Oklahoma State University, Stillwater, OK, 74078, United States.

### Clinical Samples and Processing

Five Nasopharyngeal swabs collected from human patients received at OADDL for COVID-19 testing between March 2020 – July 2020 were used in this study. Nucleic acid extraction was performed using MagMax Viral Pathogen Nucleic acid isolation kit (Thermofisher, MA, United States) as per the manufacturer’s recommended protocols. Viral presence was detected by real-time PCR using TaqPath COVID-19 Multiplex Diagnostic Solutions (Thermofisher, MA, United States). All samples had a cycle threshold value between 18 and 22.

### Genomic Sequencing

Complementary DNA (cDNA) was synthesized from extracted RNA from five clinical samples that were positive for SARS-CoV-2. cDNA was then PCR amplified using ARTIC V3 primers^2^ to obtain overlapping segments of the whole viral genome. DNA library repair (SQK-LSK-109, Oxford Nanopore Technologies, United Kingdom), Solid Phase Reversible Immobilization (SPRI) paramagnetic beads clean-up (AMPure XP, Beckmann Coulter, CA, United States) and adapter ligation and barcoding (NBD-001, Oxford Nanopore Technologies, United Kingdom) were done as per manufacturer recommendations. Libraries were then pooled and sequenced using MinION (Oxford Nanopore Technologies, United Kingdom) platform following manufacturer recommendations.

### Genome Assembly, Alignment, and Phylogenetic Analysis

Sequences obtained were assembled *de novo* using Canu (Koren et al., 2017). To further obtain a reliable consensus genome assembly, *de novo* assemblies and sequence output files were assembled to the SARS-CoV-2 reference genome Wuhan Hu-1 (GenBank ID: MN908947.3) with minimap2 (Li, 2018) and Nanopolish (Loman et al., 2015).

To assess the uniqueness of the genomes sequenced in this study, MAFFT (Katoh et al., 2009; Katoh and Standley, 2013) was used to align whole sequences of the five genomes to SARS-CoV-2 reference genome (NC_045512.2).

Gene predictions on the consensus assemblies were made using Viral Genome ORF Reader four (Wang et al., 2012) (VIGOR4) using a curated library available in the Virus Pathogen Resource (ViPR) (Pickett et al., 2012) database. Individual genes were aligned to SARS-CoV-2 Wuhan Hu-1 genome from NCBI (GenBank ID: MN908947.3) using MUSCLE aligner in MEGA-X (Kumar et al., 2018) to identify SNPs and changes in the amino acid produced by the gene.

To assess similarity to previously reported genomes, a phylogenetic analysis was made using 9072 genomes of SARS-CoV-2 from the GenBank database and the five viruses sequenced in the study. A General Time Reversible (GTR) substitution model based Unweighted Pair Group Method with Arithmetic Mean (UPGMA) alignment was constructed using MAFFT and FastTree (Price et al., 2010). Clade definitions for the sequences were identified using nine marker variants reported for classification in the GISAID database (Elbe and Buckland-Merrett, 2017).

### Structural Characterization

Crystal structures with the following codes were downloaded from protein data bank (PDB,^3^): 6VXX, 6VSB, 6M3M, and 2CJR. Computational models for the spike protein assembly were downloaded from CHARMM-GUI Archive – COVID-19 Proteins Library^4^. All measurements and analysis were performed using PyMOL opensource version 1.8.2.0^5^.

## Results and Discussion

Five clinical rRT-PCR positive samples from different periods of the pandemic in Oklahoma, United States were chosen for the study. One of the samples (Oklahoma-ADDL-1) was received during the initial stages (April 2020) of the pandemic. An increased incidence rate of the disease was observed by the end of May 2020 (OSDH-Covid-19 Tracker, 2020). Three of the samples (Oklahoma-ADDL-2,3,4) sequenced were received during this period and the last sample (Oklahoma-ADDL-5) was received 1 month (June 2020) after this period. More than 4,000X coverage was obtained at the end of sequencing for all the samples. Following *de novo* assembly with Canu, consensus genomes were obtained after reference genome assembly with nanopolish and minimap2. The genomes sequenced have been submitted to GISAID and GenBank (Table 1).

**TABLE 1:** Genome similarity of five sequenced genomes when compared to NCBI reference genome (NC_045512.2) along with total reads generated and genome coverage obtained.

**ISOLATE ID**	**GenBank ID**	**GISAID Accession number**	**Total number of reads**	**Genome coverage obtained**	**Genome similarity to NC_045512.2**
Oklahoma-ADDL 1	MT998442	EPI_ISL_535364	264,586	4,414X	99.004
Oklahoma-ADDL 2	MW000350	EPI_ISL_535361	343,075	5,844X	99.653
Oklahoma-ADDL 3	MT998443	EPI_ISL_535362	2,181,723	37,153X	99.653
Oklahoma-ADDL 4	MT998444	EPI_ISL_535363	895,721	11,904X	99.661
Oklahoma-ADDL 5 (partial)	MW000372	EPI_ISL_487231	495,578	8,260X	86.836

The five viral genome assemblies were aligned to the reference genome (SARS-CoV-2 Wuhan-Hu-1 NC_045512.2) using MAFFT and genome similarities to the reference isolate were calculated (Table 1). Most of the genomes showed more than 99% similarity. For Oklahoma-ADDL-5, only a partial sequence could be generated and hence showed a lower 86.836% similarity. This could be due to reduced amplification during amplicon generation and also due to reference assembly. Other than Oklahoma-ADDL-5, Oklahoma-ADDL-1 sequenced from a clinical sample obtained during the beginning of the pandemic in Oklahoma (April 2020) showed a lower similarity to the reference genome when compared to the other four isolates.

Genes were predicted using VIGOR4 and individual genes were aligned to their respective NCBI reference genes using MUSCLE aligner with UPGMA alignment in MEGA-X to assess mutations in the genome. Using MEGA-X visualization tool, various silent and missense mutations were detected. The missense/non-synonymous mutations detected in major genes are listed in Table 2. While non-synonymous mutations were detected in ORF1ab, ORF1a, S, N, ORF3a, ORF7, and ORF8, none were detected in Envelope (E) gene, Membrane glycoprotein (M) gene or ORF10 gene.

**TABLE 2:** Non-synonymous mutations detected and their respective amino acid changes when compared to NCBI reference genome (SARS-CoV-2, Wuhan Hu-1, NC 045512.2).

**Mutation observed (nucleiotide position)**	**Gene**	**Genomes mutation detected in**	**Mutation type**
P3371S (C10376T)	ORF1ab	Oklahoma-ADDL-5	Transition
T4412A (A13498G)	ORF1ab	Oklahoma-ADDL-4	Transition
P4715L (C14408T)	ORF1ab	Oklahoma-ADDL-1,2,3,4	Transition
A6269S (G19069T)	ORF1ab	Oklahoma-ADDL-2,3	Transversion
D614G (A23403G)	S	Oklahoma-ADDL-1,2,3,4,5	Transition
G1167V (G25062T)	S	Oklahoma-ADDL-4	Transversion
Q57H (G25563T)	ORF3a	Oklahoma-ADDL-1	Transversion
G96R (G28179C)	ORF8	Oklahoma-ADDL-2,3	Transition
T28I (C27476T)	ORF7b	Oklahoma-ADDL-5	Transversion
S194L (C28854T)	N	Oklahoma-ADDL-4	Transition
R203K (G28881A; G28882A)	N	Oklahoma-ADDL-2,3,5	Transition
G204R (G28883C)	N	Oklahoma-ADDL-2,3,5	Transversion

Non-synonymous mutation at amino acid location 81 (C→T; Arginine → Cysteine), which codes for nsp2 (non-structural protein)was present in all the genomes sequenced in the study. The potential implication of this mutation is unknown. nsp2 along with nsp3 are known to play major roles in pathogenesis (Angeletti et al., 2020) of SARS-CoV-2 in humans. A few other possibly novel and previously reported non-synonymous mutations in ORF1a and ORF1ab were also identified. A previously reported mutation in the ORF1ab gene P4715L (Banerjee et al., 2020b) (Proline to Leucine) was recorded alongside novel mutations at various amino acid locations. P4715L mutation has been implicated to play a major role in interaction with other proteins that regulate RNA Dependent RNA polymerase activity. P3371S (Proline to Serine) mutation in ORF1ab and ORF1a was detected in Oklahoma-ADDL-5. Oklahoma-ADDL-4 carried mutation T4412A (Threonine to Alanine) in ORF1ab, while Oklahoma-ADDL-2 and 3 carried mutation A6269S (Alanine to Serine). ORF1ab has multiple functions including RNA dependent RNA polymerase activity, helicase activity, Fe–S cluster binding, Zn^–^ binding activity, methyltransferase activity (Graham et al., 2008) etc. Functional implications of these mutations are still unknown and further studies to understand functional changes caused by these mutations may aid in better understanding the pathogenesis of the viral isolates found in Oklahoma.

Non-synonymous mutations were also found in S, ORF3a, ORF7b, ORF8, and N gene (Table 2). A previously reported mutation in S gene – D614G (Laha et al., 2020) (Aspartate to Glycine), was identified in all the genomes sequenced. The D614G mutation has been reported to cause a decrease in PCR cycle thresholds, suggestive of higher upper respiratory tract viral load (Grubaugh et al., 2020; Korber et al., 2020) in the host. A previously reported deleterious variation in the protein expressed from ORF3a, Q57H (Issa et al., 2020; Laha et al., 2020), was recorded in Oklahoma-ADDL-1, the genome isolated at the beginning of the pandemic in Oklahoma. This mutation was not recorded in genomes isolated at other times in the state. The N gene also carried other previously reported mutations (Table 2). Oklahoma-ADDL-4, carried a non-synonymous mutation S194L (Banerjee et al., 2020a) while Oklahoma-ADDL 2,3,5 carried R203K and G204R (Laha et al., 2020).

A few possible novel mutations were also identified in S, ORF3a, ORF7b, and ORF8 genes (Table 2). A mutation in the S gene, G1167V (Glycine to Valine) was identified in Oklahoma-ADDL-4. T28I in ORF7b gene, a non-synonymous mutation (Threonine to Isoleucine) in the genome Oklahoma-ADDL-5 and in ORF8, G96R non-synonymous mutations resulting in Glycine to Arginine were noted in Oklahoma-ADDL-2 and Oklahoma-ADDL-3. These mutations in the genome indicate presence of multiple variations of the virus in Oklahoma.

A phylogenetic tree was constructed using 9,072 genomes available in the NCBI repository. The nearest neighbors to the genomes were found to be isolates reported from San Diego and Atlanta in United States and from Greece and Australia. While the Oklahoma-ADDL-1 and 5 were phylogenetically more related to isolates reported from Australia, Oklahoma-ADDL-2 and 3 were phylogenetically related to isolates reported from Greece and Atlanta, United States. Oklahoma-ADDL-4 was phylogenetically related to the isolate reported from San Diego United States. Oklahoma-ADDL-1 was identified to be in clade-GH and Oklahoma-ADDL-2,3,4,5 were identified to be in clade GR as per annotations of different marker variants in the GISAID database. The presence of multiple isolates within the Oklahoma sheds light on contagious nature of these viruses.

### Functional Significance of S Gene Mutations

**FIGURE 1:**
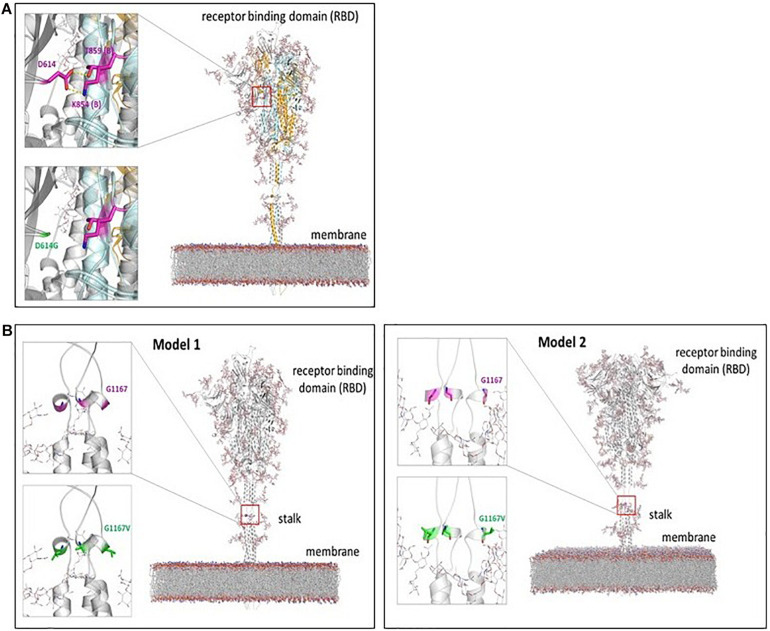
Mutations in the Spike protein. **(A)** Change in interactions within spike protein due to D614G mutation: The mutation is located in the RBD of the spike protein. Model based on groups Croll, Seok, and Im is depicted (Choi et al., 2020; Woo et al., 2020). This model shows glycosylated protein embedded in a membrane; the spike protein is homo-trimer, with three protein chains shown in gray, cyan, and light orange. The location of Asp 614 is indicated by red square in the full model. The zoomed in view on the left shows the interacting residues. The side chain of Asp 614 from one monomer (gray chain) makes hydrophilic interactions with residues Lys 854 and Thr 859 from adjacent monomer (cyan chain indicated by **(B)** in the residue labels). Additional hydrophobic interactions with Val 860 and Leu 861 have also been suggested. The mutation D614G causes loss of all these interactions. **(B)** Location of G1167V mutation in spike protein: Two alternate computational models are shown, as the structure of the full-length spike protein has not been solved so far. These two alternate models indicate that the Gly 1167 is located in the stalk region between the RBD and the transmembrane region. The position is predicted to be a part of short alpha helical region. The mutation G1167V may affect the alpha helical region as well as the bending motion of the stalk region.

D614G mutation in S gene, which corresponds to changes in the spike protein, has already been reported widely in the literature (Grubaugh et al., 2020; Korber et al., 2020; Laha et al., 2020). As shown in Figure 1A, crystal structure and the molecular models of spike protein assembly developed by several groups indicate that the residue Asp 614 makes contact with several other residues in the trimeric spike protein assembly (Choi et al., 2020; Walls et al., 2020; Woo et al., 2020; Wrapp et al., 2020). Most noticeably, the sidechain of Asp 614 makes hydrophilic contacts with residues Lys854 and Thr859 (of the adjacent monomer of spike protein). Additional hydrophobic interactions with residues Val 860 and Leu 861 have also been reported (Isabel et al., 2020). The mutation to Gly 614 removes all these side chain interactions. The functional relevance of this mutation is currently being explored in a number of ongoing investigations (Choi et al., 2020; Gobeil et al., 2020), particularly in relation to conformational dynamics of the receptor binding domain (RBD).

The mutation G1167V in S gene is proposed to be located in the stalk region of spike protein. A structure of full-length spike protein is currently not available. Models prepared by computational groups (Choi et al., 2020; Woo et al., 2020) (Figure 1B) have indicated that this mutation is located in the heptad repeat (HR) linker region between RBD of the trimeric spike protein and the transmembrane (TM) region. Two alternate computational models developed by Woo et al., 2020 and Choi et al., 2020 (Choi et al., 2020; Woo et al., 2020) (shown in Figure 1B), indicate that Gly 1167 is possibly part of a short alpha helical region. The mutation to Val 1167 introduces a relatively bulkier side chain, and its effect on the secondary structure is currently unknown. However, this mutation is located in the stalk region, which is proposed to affect the bending motions of the stalk causing large movements of the RBD. The conformational flexibility of spike protein and movements of the RBD have already been suggested to be linked to its function of binding to ACE2 (Gobeil et al., 2020).

### Functional Significance of N Gene Mutations

**FIGURE 2:**
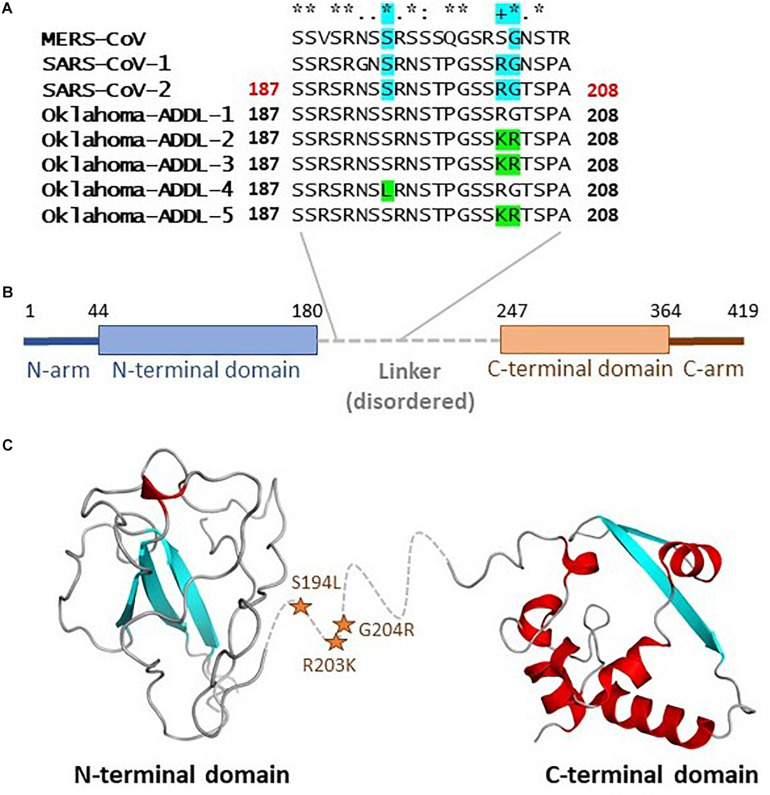
Mutations in the nucleocapsid protein: The N gene product plays a role in RNA packaging and viral release. **(A)** The mutations S194L, R203K, and G204R are located in the highly conserved linker region as indicated in panel. **(B)** Various regions in the protein. **(C)** The structure of full-length protein is not available, structure of NTD (based on 6M3M chain A) and CTD (based on 6WJI chain A) are depicted with linker region indicated with the dashed line in the middle. The location of the mutations is indicated by stars.

The N gene product is reported to be the nucleocapsid protein which plays a role in RNA packaging and viral particle release (Zeng et al., 2020). The crystal structure of the full length nucleocapsid protein has not been solved so far, however, it is proposed to consist of several regions (Figure 2) including N-arm, N terminal domain (NTD), linker region, C-terminal domain (CTD) and the C-tail. The structures of NTD and CTD from SARS-CoV-2 has been solved. N gene mutations idenfied in this study (S194L, R203K, and G204R) are located in the region 180–247, which is suggested to be a flexible linker region that lacks organized structure. Based on small angle X-ray scattering (SAXS) studies, it is proposed that this region is extended and may contain some residual secondary structure (Zeng et al., 2020).

The site of two of these mutations (S194 and G204) are fully conserved between MERS-CoV, SARS-CoV-1, and the reference SARS-CoV-2 sequence, while the site of third mutation (R203) is conserved in the SARS-CoV-1 and the reference SARS-CoV-2 sequence. The conservation of these residues between different viruses may be an indication of their functional role. It is widely discussed that protein regions that lack secondary structure become structured in presence of proper binding partners or may be involved in signaling mediated by flexibility and conformational sampling. Future studies would be important in characterizing the functional relevance of these mutations.

## Conclusion

We sequenced five SARS-CoV-2 genomes from clinical samples collected from Oklahoma, United States between March and July 2020. Genome assembly and annotation studies identified several new mutations as well as previously reported mutations. Notably, presence of D614G mutation in the S gene was found in all the isolates. Detection of multiple mutations in the viral genomes collected from a narrow geographic region within a few months of the pandemic underscores the ability of SARS-CoV-2 to undergo rapid genomic alterations. Further studies are needed to better understand if these mutations can potentially influence host susceptibility, pathogenicity and virulence. Phylogenetic analysis of the viral genomes revealed high similarities with isolates reported from Australia, Greece and United States (Atlanta and San Diego), indicating possible multiple introductions to the state. Preliminary characterization based on available structural information of the SARS-CoV-2 proteins indicates that the mutations in S gene possibly influences conformational flexibility and motion of the spike protein, and the mutations in N gene are associated with disordered linker region within the nucleocapsid protein. In the future, mass sequencing of clinical isolates is needed to comprehensively identify genomic variations of SARS-CoV-2 in specific geographic locations.

## Data Availability Statement

The datasets presented in this study can be found in online repositories. The names of the repository/repositories and accession number(s) can be found below: https://www.ncbi.nlm.nih.gov/genbank/, MT 998442MW 000350 MT 998443 MT 998444 MW000372 https://www.gisaid.org/, EPI_ISL_535364, EPI_ISL_535361, EPI_ISL_535362, EPI_ISL_ 535363, and EPI_ISL_487231.

## Ethics Statement

This study was approved by the Institutional Review Board (Application Number: IRB-20-357) at Oklahoma State University, Stillwater OK 74078, United States.

## Author Contributions

SN and AR performed the sequencing. SN and JR performed the bioinformatics analysis. All the authors helped with sample collection. AR, AK, SM, JM, and JS helped with preparing and reviewing the manuscript. All authors contributed to the article and approved the submitted version.

## Conflict of Interest

The authors declare that the research was conducted in the absence of any commercial or financial relationships that could be construed as a potential conflict of interest.

## Footnotes

1 www.epicov.org

2 https://github.com/artic-network/artic-ncov2019/tree/master/primer_schemes/nCoV-2019/V3

3 https://www.rcsb.org/

4 http://www.charmm-gui.org/?doc=archive&lib=covid19

5 http://pymol.org/
